# Adaptation response of *Pseudomonas fragi* on refrigerated solid matrix to a moderate electric field

**DOI:** 10.1186/s12866-017-0945-2

**Published:** 2017-02-10

**Authors:** Wenbo Chen, Honghai Hu, Chunjiang Zhang, Feng Huang, Dequan Zhang, Hong Zhang

**Affiliations:** 1 Institute of Food Science and Technology, Chinese Academy of Agricultural Sciences/Key Laboratory of Agro-Products Processing, Ministry of Agriculture, Beijing, 100193 People’s Republic of China; 2 College of Staple Food Technology, Chinese Academy of Agricultural Sciences, Institute of Food Science and Technology, Harbin, 151900 People’s Republic of China

**Keywords:** Adaptation response, Moderate electric field, Nutrient agar plates, Psychrotrophic microorganisms, *Pseudomonas fragi*, Solid cellular foodstuffs

## Abstract

**Background:**

Moderate electric field (MEF) technology is a promising food preservation strategy since it relies on physical properties—rather than chemical additives—to preserve solid cellular foods during storage. However, the effectiveness of long-term MEF exposure on the psychrotrophic microorganisms responsible for the food spoilage at cool temperatures remains unclear.

**Results:**

The spoilage-associated psychrotroph *Pseudomonas fragi* MC16 was obtained from pork samples stored at 7 °C. Continuous MEF treatment attenuated growth and resulted in subsequent adaptation of M16 cultured on nutrient agar plates at 7 °C, compared to the control cultures, as determined by biomass analysis and plating procedures. Moreover, intracellular dehydrogenase activity and ATP levels also indicated an initial effect of MEF treatment followed by cellular recovery, and extracellular β-galactosidase activity assays indicated no obvious changes in cell membrane permeability. Furthermore, microscopic observations using scanning and transmission electron microscopy revealed that MEF induced sublethal cellular injury during early treatment stages, but no notable changes in morphology or cytology on subsequent days.

**Conclusion:**

Our study provides direct evidence that psychrotrophic *P. fragi* MC16 cultured on nutrient agar plates at 7 °C are capable of adapting to MEF treatment.

## Background

Alternating electric fields stall the planktonic growth of several prokaryotic microorganisms, such as *Escherichia coli*, *Lactobacillus acidophilus, Staphylococcus aureus, Bacillus cereus, Pseudomonas putida,* and *P. fluorescens* [1–6], leading to its recent application in food preservation [7]. The effectiveness of alternating electric field technology in preventing spoilage can be easily assessed by analyzing the amount of proteins eluted from cellular samples [7]. Alternating electric fields are generally used at either high intensity or high frequencies [1, 3, 4, 7, 8], and the possible applications of moderate electric fields (MEFs, typically <1–100 V/cm) at low frequencies (1–100 Hz) have been far less studied [9]. For example, planktonic *L. acidophilus* exhibits a decreased lag time following treatment with a MEF [6, 9], causing a marked increase in bacteriocin yield [6]. However, few studies have investigated the application of MEF as a food preservative.

Refrigeration is a common food preservation strategy that extends the shelf life of food products by decreasing microbial growth rates and slowing the physical and chemical reactions responsible for spoilage [10–12]. However, microbial species adapted to the temperatures ranging from 0 °C to 22 °C (*e.g.*, psychrotrophs) outcompete other microorganisms and dominate the microbial population present on food products during refrigerated storage [12–14], and thus are the primary contributors to food spoilage at refrigeration temperatures [15–19].

*Pseudomonas fragi* is a psychrotrophic species responsible for the majority of spoilage of meat stored aerobically at refrigeration temperatures [17]. *P. fragi* is more widely distributed than other pseudomonads, and has been isolated from water, soil, plant materials, and other natural media [17, 20–22]. This species grows well at temperatures ranging from 2 °C to 35 °C [21]. The prevalence and growth conditions of *P. fragi* contribute to its successful proliferation on foods, especially fresh meat [12, 17, 21, 23, 24], and the strict chill chain applied during fresh meat production from slaughtering to final distribution selectively favors its development [21, 25]. Moreover, *P. fragi* is suggested to promote the growth and survival of several foodborne pathogens, such as *S. aureus* and *Listeria monocytogenes* [26, 27]. Therefore, information about *P. fragi* growth and biological responses to preservation conditions during food storage is urgently needed to improve food safety.

MEF technology may provide a means to control microbial growth on food products where refrigeration fails to do so. However, organisms may develop adaptive responses to continuous MEF treatment, similar to how *P. fragi* has adapted to cooler temperatures [21]. Thus, the aim of this work was to investigate the biological responses of psychrotrophic microorganisms to continuous MEF treatment by characterizing the adaptive response to MEF treatment in *P. fragi*. Additionally, we examined cell structural changes induced by MEF treatment using electron microscopy.

## Methods

### Meat sample preparation and storage

Pork samples were prepared as described by Ercolina et al. [24] with some modifications. Pork muscle (*Longissimus dorsi*) obtained from a slaughterhouse was cut into similar-sized pieces (2 × 2.5 × 3 cm) and packed in sterile stomacher bags (Labplas, Inc., Sainte-Julie, Quebec, Canada). The prepared samples were then stored aerobically at 7 °C for at least 5 days and used as sources for the isolation of psychrotrophs [12, 26].

### Bacterial strain isolation and identification

A meat sample (25 g) was aseptically weighed and homogenized in 225 mL of peptone saline (0.85% NaCl and 0.1% peptone in distilled water) at room temperature (22–25 °C) [27–29]. Serial dilutions were prepared with the homogenized meat sample and 100 μL aliquots of the appropriate dilutions were spread in triplicate on nutrient agar plates (Luqiao Co., Beijing, China) and incubated for 5 days at 7 °C [24]. Colonies present on the countable plates were randomly picked and further purified by repeated streaking. Finally, these purified cultures were maintained as glycerol stock culture and stored at -80 °C [30]. These isolates were also deposited on nutrient agar slants in the dark at 4 °C [31].

The isolates were identified by 16S rDNA sequencing carried out by Sangon Biological Co., Ltd. (Shanghai, China).

### MEF system

**Fig. 1:**
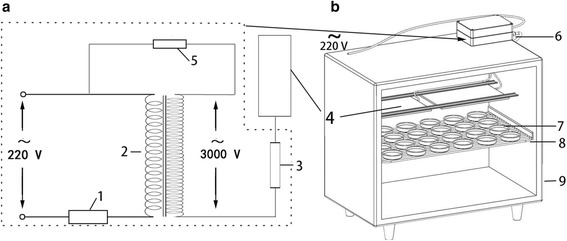
Schematic diagrams of the alternating electric field (**a**) and a refrigerator equipped with the alternating electric field (**b**). 1: input controller; 2: transformer; 3: output controller; 4: discharge plate; 5: feedback control circuit; 6: alternating electric field generator; 7: petri dishes; 8: sample holder; 9: refrigerator

The MEF system used in this study was designed and patented by Takayuki et al*.* [32]. The system consisted of an input controller, transformer, feedback control circuit, output controller, and discharge plate. The transformed voltage was exerted on the discharge plate via the output controller, which could make the discharge plate generate a wide range of electric fields [32]. The waveform of the output voltage measured with an oscilloscope (UTD2102CEX, Uni-Trend) and a high-voltage prober (P6015A, Tektronix) is a sine wave with a 9.2 kV maximum voltage (peak-to-peak). The frequency of the output voltage is 50 Hz (as described in [6, 9]) and identical to that of the input voltage. Other parameters are identical to those described in the patent [32]. The schematics of this apparatus and the refrigeration system are shown in Fig. 1.

### Cultivation on nutrient agar plates

Microbial isolates were grown on nutrient agar plates because of its extensive use in microbiological studies and relatively definite components. Fresh bacterial cultures were prepared from a glycerol stock culture. After cultivation at 7 °C for 3 days, a single colony on nutrient agar plate was randomly chosen to prepare a bacterial suspension in sterile peptone saline. After serial dilution, 100 μL of the appropriate dilutions (approx. 3,000 CFU/mL) were spread onto nutrient agar plates and were incubated at 7 °C in either a control refrigerator or a refrigerator equipped with the MEF system. MEF-treated samples were placed on holders and continuously exposed to MEF (20 V/cm) throughout the cultivation period (Fig. 1b).

### Microbial growth analysis

Microbial growth of MEF-treated and control cultures was first evaluated by observing the full biomass development on nutrient agar plates as described by Ercolini et al [24]. Because of the low growth rate at cold temperatures, only cultures incubated for 3, 4, and 5 days were used.

Culture growth was also determined by monitoring the population using plating procedures. Plated cultures were suspended in 4 mL of peptone saline [22], serially diluted, replated on nutrient agar, and then colonies were counted after incubation at 7 °C for 3 days [16, 33]. Three plates were prepared for daily test for each group.

### Dehydrogenase activity assay

Dehydrogenase activity was assayed by MTT (3-(4, 5-dimethylthiazol-2-yl)-2, 5-diphenyltetrazolium bromide; Beyotime Biotechnology, Jiangsu China) analysis described by Wang et al. [34] with slight modifications. Briefly, 3-, 4-, or 5-day microbial cultures were diluted to an OD_600_ of 0.2 in fresh nutrient broth. A 200 μL aliquot of the cell suspension was then mixed with 20 μL MTT solution (5 mg/mL in sterilized water) and incubated for 20 min at 25 °C. After centrifugation at 12,000 rpm for 1 min, pelleted cell-formazan crystal complexes were transferred into glass test tubes using 2.5 mL of dimethyl sulfoxide (DMSO, Sinopharm, China). The OD_550_ values of the mixtures were measured with a Universal Microplater Reader (Molecular Devices, Sunnyvale, CA) at 10–15 min. Three plates per group were prepared for each test.

Dehydrogenase activity is expressed in MTT reduction units (MRU). One MRU is defined as an A_550_ value of 1.0 produced by the dissolved formazan crystals from cells incubated in LB medium at 7 °C in 20 min [34].

### Intracellular ATP levels determination

Intracellular ATP levels were measured using a firefly luciferase-based bioluminescence ATP Assay Kit (Beyotime Biotechnology). Cell suspensions prepared from 1 plate was diluted to an OD_600_ of 0.2 and centrifuged at 12,000 rpm for 5 min at 4 °C. The obtained cell pellets were used to prepare intracellular ATP solution according to the supplier’s instructions. Cells were mixed with lysis solution and boiled for 2 min to release ATP and to inactivate endogenous ATPase [35, 36]. Then, 20 μL ATP extraction and 100 μL ATP detection solutions were mixed to prepare the luminescent reaction solution.

Luminance (RLU) was recorded using Hidex plate Chameleon™ V. (Finland) and linearly related to the ATP concentration.

### β-Galactosidase activity assay

β-Galactosidase activity was measured according to Gobinath and Prapulla [37] with some modifications. Cultures grown on 1 agar plate supplemented with 1% lactose were suspended in 4 mL phosphate buffered saline (PBS). The OD_600_ of these cultures was measured and the suspension were centrifuged at 15,000 rpm for 5 min. β-Galactosidase activity in the resulting supernatant was determined by mixing 1 mL supernatant and 0.2 mL ONPG (4 mg/mL, *o*-nitrophenyl-β-D-galactopyraniside). The reaction was terminated after incubation at 25 °C for 20 min by adding 0.5 mL 1 M Na_2_CO_3_. The optical density of the reaction solution was recorded at 420 nm (OD_420_) and 550 nm (OD_550_), as previously described [38]. β-Galactosidase activity was calculated in Miller Units that describe the change in OD_420_/min/mL of cells/OD_600_ [39].

### Microscopic observations

Control and MEF-treated cultures were observed using a scanning electron microscope (SEM; S570, Hitachi, Japan) and a transmission electron microscope (TEM; H-7500, Hitachi). Cultures grown on nutrient agar plates for 3 days were rinsed 3 times with sterilized distilled water. After centrifugation at 15,000 rpm for 5 min, cell pellets were suspended and fixed in glutaraldehyde (3% aqueous solution). Other steps employed to prepare samples for SEM and TEM were carried out according to the methods described by Racyte and Zituni [1, 40].

### Statistical analysis

The effect of MEF on *P. fragi* growth on nutrient agar plates at refrigeration temperature was evaluated by three independent experiments. The obtained data were processed with the data module of the Statistical Package for Social Science (IBM SPSS Statistics 19) for Windows and assessed with *t*-tests. A significant difference between the treated and control groups was defined as a *p* value < 0.05.

## Results and Discussion

Isolation and identification of psychrotrophic bacteria from meat samples

After repeated isolation and purification, a total of 25 isolates were obtained from the pork samples. All purified isolates formed visible colonies within 3 days at 7 °C, indicating their adaptation to the refrigeration temperature. These colonies produced the intense fruity odor that is characteristic of pseudomonads, suggesting that these isolates might be *P. fragi* [17, 21].

16S DNA sequencing revealed that the 25 isolates all shared 99% identity with *P. fragi* isolate 7U (Accession No.: LK391544) [41]. The 16S rDNA sequence was deposited in GenBank as KT443784 and the isolate designated as *P. fragi* MC16. These sequencing results indicate that *P. fragi* is primarily responsible for the spoilage of fresh meat stored at refrigeration temperatures. Although many microorganisms are associated with meat spoilage, few species are able to grow at the low temperatures used to preserve perishable foods [12, 15, 16, 18, 22, 24]. Several *Pseudomonas* species (*e.g*., *P. fragi, P. lundensis, P. fluorescens,* and *P. putida*) have been frequently identified as major spoilage agents in fresh pork, beef, and mutton [12, 17, 21, 23, 24, 42], but only *P. fragi* was isolated from meat samples in this study. Sample storage and processing conditions might have favored *P. fragi* growth by eliminating other spoilage-related species [22]. Additionally, the biotype diversity of *P. fragi* might contribute to this result because different molecular types of *P. fragi* might behave similarly as meat spoilers [21].

### Growth of *P. fragi* MC16 on nutrient agar plates

**Fig. 2:**
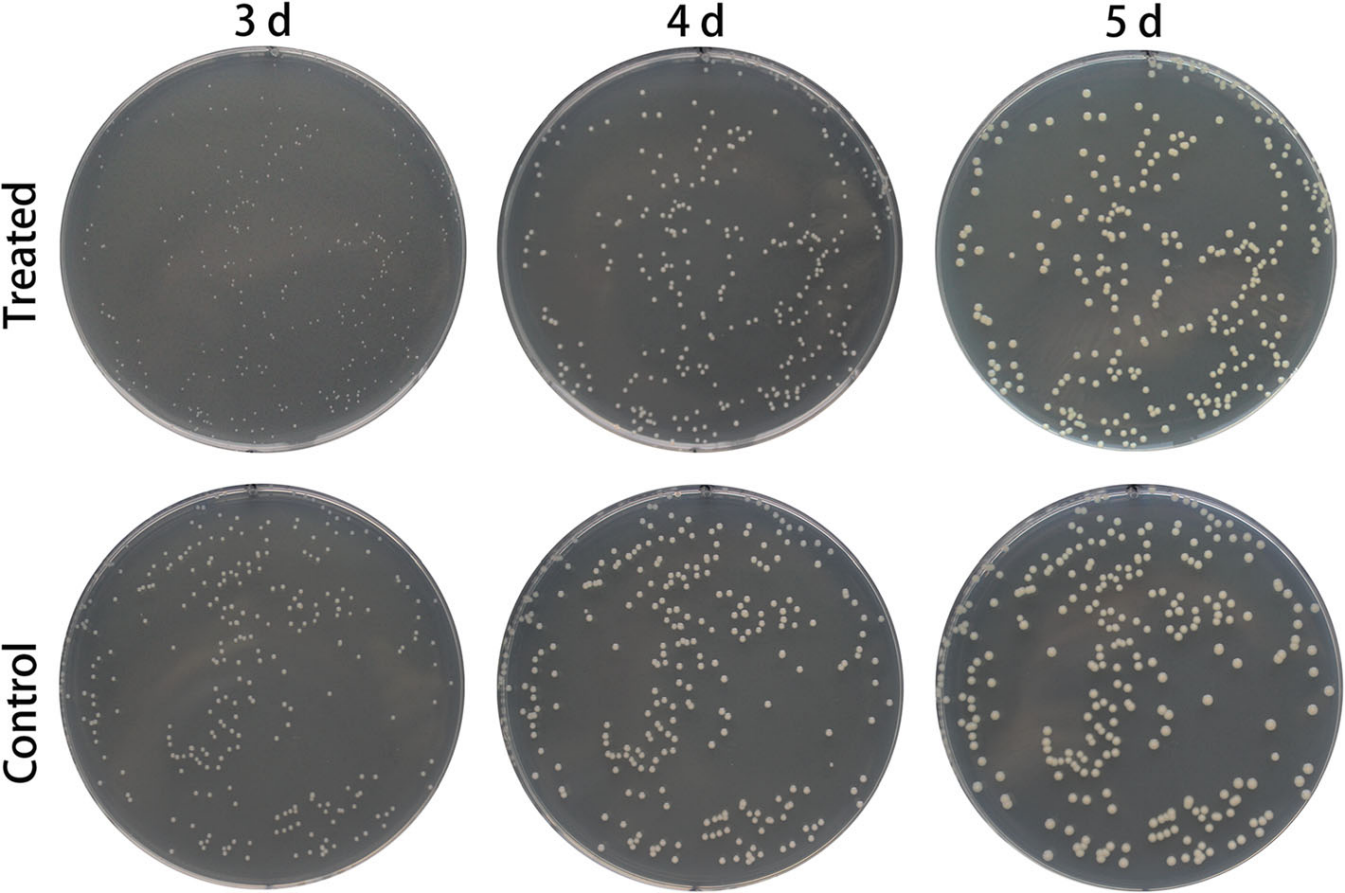
*P. fragi* MC16 biomass development on nutrient agar plates for different incubation durations. An identical petri dish was employed on different days for each group

Control and MEF-treated cultures could be clearly differentiated by observing biomass development. MEF-treated colonies were smaller than untreated colonies after 3 days of growth (Fig. 2). However, the size difference gradually decreased after 4 and 5 days of incubation.

**Fig. 3:**
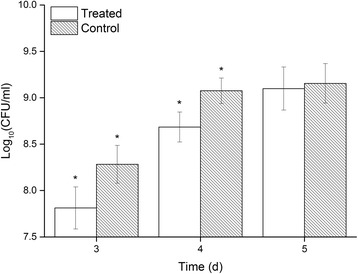
Changes in total viable counts of *P. fragi* MC16 grown on nutrient agar plates for different times at 7 °C. * indicates a significant difference from the control group, *p* < 0.05

Total viable counts of the control and MEF-treated cultures were determined to quantify the effect of MEF treatment on the growth of *P. fragi* MC16. Total viable counts of the treated and untreated cultures were significantly different (*p* < 0.05) after 3 and 4 days of growth (Fig. 3). Similar to the qualitative biomass development assessments, no significant difference in quantitative biomass between the two groups was found after 5 days of incubation. These results are consistent with a previous study by Loghavi [6], indicating that *P. fragi* MC16 responds differently to MEF exposure during different growth phases.

Changes in the susceptibility of *P. fragi* MC16 on nutrient agar plates to MEF treatment can be attributed to limited nutrient diffusion in the solid matrix and culture age. Previous studies suggested that MEF treatment at lower frequencies could cause temporary, non-lethal membrane permeabilization [9, 43]. For cells grown in liquid medium, increased permeability enhances nutrient diffusion across cell membranes and reduced lag phases are observed [6]. However, nutrient diffusion in solid medium is relatively low. As a result, temporary permeabilization on nutrient agar plates causes sublethal damage rather than rapid nutrient uptake. Therefore, retarded cell growth was observed in this study. Additionally, older cultures have decreased protein to lipid ratios in membranes [9, 43], causing decreased membrane fluidity and susceptibility to external MEF treatment. Thus, biomass recovered later during incubation.

### The effect of MEF treatment on *P. fragi* MC16 metabolic activity

**Fig. 4:**
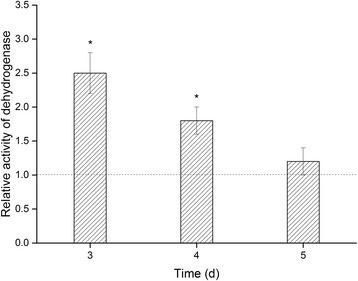
The effect of MEF on intracellular dehydrogenase activity. Dehydrogenase activity is expressed in relative units as the ratio of dehydrogenase activity of the treated group to the control group. Error bars represent standard error. The dashed line indicates a ratio of one (where values of the treated group are equal to the control group). * indicates significant difference from the control group, *p* < 0.05

MEF is an environmental stress to which microorganisms can adapt, similar to cold, heat, and pressure [3, 36]. Microorganisms can modulate their metabolism to cope with sublethal stressors [44, 45] and may enter different growth phases that alter their susceptibility to the stress [6, 9]. Therefore, investigating metabolic changes is one method to characterize microbial stress responses. Dehydrogenases are key enzymes involved in the oxidative activities of various organisms, thus dehydrogenase activity reflects the metabolic state of the organism [36, 46]. We measured intracellular dehydrogenase activity during MEF treatment to characterize the metabolic state of *P. fragi* MC16. MEF-treatment significantly increased intracellular dehydrogenase activity for treated cultures after 3 and 4 days (*p* < 0.05; Fig. 4). Intracellular dehydrogenase activity of MEF-treated cultures after 3 and 4 days was 2.5- and 1.8- times higher than the control groups, respectively. However, dehydrogenase activity decreased after 5 days of treatment such that the enzyme activity in MEF-treated cultures was only 1.2-fold higher than in control cultures. This difference was not significant.

Increased dehydrogenase activity is generally associated with the energy-consuming microbial stress response [44, 46]. This response has been previously observed in *S. aureus* exposed to high osmolarity or high pressure [47]. We found that increased dehydrogenase activity was readily observed when *P. fragi* cultures were treated with MEF. Due to the sublethality of MEF, microorganisms exposed to this field must repair injured membranes [48]. Additionally, MEF treatment may impact the conformation of cellular proteins [9]. To restore normal protein conformations and survive these conditions, microorganisms must modulate gene expression and modify metabolism to engage adaptive cellular mechanisms [49]. All metabolic changes induced by MEF treatment reflected higher metabolic investment, which is generally characterized by increased dehydrogenase activity and ATP level [36]. These metabolic changes may have diminished after 5 days of treatment and incubation because the cells activated adaptive mechanisms and modulated gene expression to manage the stressor.

### The effect of MEF on intracellular ATP concentration

**Fig. 5:**
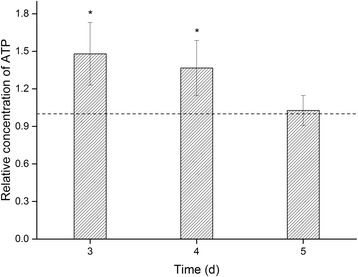
The influence of MEF on intracellular ATP concentration in *P. fragi*. Values are presented as ratios of ATP concentration (RLU/OD _600_) in treated cultures relative to control cultures. The dashed line indicates a ratio of one (where means values of treated cultures are equal to control cultures). * indicates a significant difference from the control cultures (*p* < 0.05)

ATP is a direct participant in many cellular metabolic processes [36]. Therefore, changes in ATP concentration may characterize the metabolic state during MEF treatment more specifically than changes in dehydrogenase activity. As shown in Fig. 5, MEF treatment increased intracellular ATP content. ATP concentrations in cultures treated for 3, 4, and 5 days were 1.5-, 1.4-, and 1.1-times higher than control cultures, respectively. Other forms of environmental stress (*e.g*., heat, pressure, starvation, pH, and irradiation) have been previously reported to increase ATP concentration in microorganisms [50], suggesting that MEF-treatment causes a similar cellular response as these stressors. The concentration of intracellular ATP in MEF-treated cells decreased to the level of the controls after 5 days of exposure, indicating metabolic adaptation to the MEF treatment. This trend in intracellular ATP content reflects similar findings in biomass development and intracellular dehydrogenase activity of the treated cultures over time.

### The effect of MEF on inner membrane permeability

β-Galactosidase is a large, tetrameric protein [39]. Because of its high molecular weight, presence of extracellular β-galactosidase is generally associated with a loss of cell membrane integrity [51]. Thus, the activity of extracellular β-galactosidase produced by MEF-treated and untreated cultures was quantified to indicate cell membrane alterations over time.

**Fig. 6:**
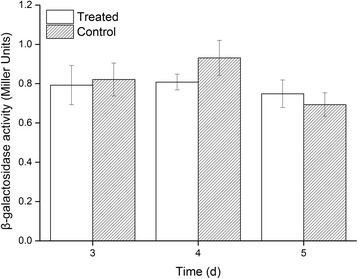
Extracellular β-galactosidase activity of the treated and untreated cultures grown on nutrient agar plates for different times

Extracellular β-galactosidase activity levels in treated and untreated cultures were 0.79 and 0.80 Miller Units, respectively, after 3 days of cultivation. Extracellular β-galactosidase activity levels were not significantly different between treated and untreated cultures at 3, 4, or 5 days of cultivation (Fig. 6).

These results suggest that MEF treatment did not increase cell membrane permeability, since permeabilized membranes would allow intracellular β-galactosidase to leak into extracellular space. Membrane permeabilization via MEF has been reported in several studies [6, 9, 48]; however, permeabilization is reversible and membrane integrity can be quickly reestablished [48]. Accordingly, the low levels of extracellular β-galactosidase activity observed in this study may indicate that cellular membranes were reestablished too rapidly to detect major changes. In addition, the MEF system used in this study was designed to maintain the freshness of solid cellular foods, such as meat, fish, fruits, and vegetables [32]. The MEF system was not designed to inactivate microorganisms by forming irreversible membrane permeabilization, since inactivation would compromise the freshness and nutrition of the cellular foods that also have cell membranes [7]. Moreover, the intensity of the EMF used in this study was lower than 30 V/cm, ruling out the possibility of electroporation that was reported to occur at field intensities around 1,000 V/cm [3, 52].

### Morphological changes of *P. fragi* MC16 induced by MEF

Intact cell morphology and cytology are essential for the normal growth and multiplication of microorganisms, thus alterations in microbial morphology and cytology reflect cell viability [5, 40, 51]. Microscopic observations of cultures treated for different incubation periods were conducted using SEM and TEM.

**Fig. 7:**
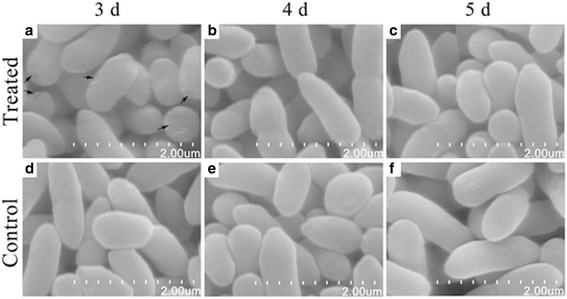
Scanning electron microscope observations of *P. fragi* MC16 grown for different times. **a**–**c** Treated samples; **d**–**f** Control samples. Arrows indicate cell surface indentations

SEM observations illustrated changes in cell morphology (Fig. 7). Cultures treated with MEF for 3 days were largely intact, but showed surface indentations (indicated with arrows in Fig. 7a). Cellular debris derived from cell disintegration has been reported by previous literature [5, 40], but was not observed in this study. These results support the findings of the extracellular β-galactosidase activity: MEF treatment appears to have limited impact on microbial cell membranes. Microbial cells treated with MEF for 4 and 5 days could not be differentiated from control cells (Fig. 7e and f), which were characterized by smooth cell surface and uniform cell shapes. The cell morphologies of control and MEF-treated cells after 3 days of treatment may indicate that the treated cells adapted to the MEF exposure. SEM examinations of untreated cultures showed no changes in cell morphology throughout the process (Fig. 7d, e, and f).

**Fig. 8:**
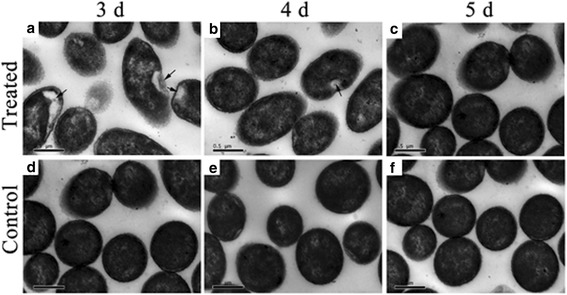
Transmission electron microscope observations of *P. fragi* MC16 grown for different times. **a**–**c** Treated samples; **d**–**f** Control samples. Arrows indicate agglomerations of intracellular contents

TEM observations of MEF-treated cultures showed agglomerations of cytoplasmic materials, vacuole formations, and depressed cell membranes (indicated with arrows in Fig. 8a and b). These cellular observations are consistent with cell damage induced by continuous ultrasound in *E. coli* ATCC 11775 [53]. Nevertheless, these changes gradually disappeared when the exposure time was extended to 5 days (Fig. 8c). At this point, TEM observations of treated and control cultures (Fig. 8f) were almost identical, suggesting that the cells had completely adapted to the MEF treatment. Control cells showed regular shape and morphology, with the cytoplasm filling the entire cell envelope (Fig. 8d, e, and f).

Summarizing the observations on morphology and cytology, cultures at early growth stages might be susceptible to continuous MEF exposure; however, they subsequently recovered from these injuries as the treatment progressed. These results indicate the microbial adaptation to the MEF treatment and support previous findings that the bacterial stress response supports adaptation to adverse environments [49].

## Conclusions

MEF application is a novel technology for preserving solid cellular foods. The technology can cause adverse morphological and cytological changes to microorganisms growing on a solid matrix during early cell growth stages. However, the impact of MEF application gradually weakens as cells potentially responded to the stress via modified metabolism and gene expression changes. Therefore, future research exploring the adaptive mechanisms to MEF is required before this technology can be improved to further extend the shelf life of solid cellular foodstuffs and reduce food losses caused by microorganisms.

## Abbreviations

ATP: Adenosine triphosphate
DNA: Deoxyribonucleic acid
MEF: Moderate electric field
MRU: MTT reduction units
MTT: 3-(4, 5-dimethylthiazol-2-yl)-2, 5-diphenyltetrazolium bromide
OD: Optical density
ONPG: o-nitrophenyl-β-D-galactopyraniside
RLU: Relative luminance unit
SEM: Scanning electron microscope
TEM: Transmission electron microscope
